# Exosome therapy for flap and skin graft survival: A systematic review and meta-analysis of preclinical evidence

**DOI:** 10.1016/j.jpra.2026.05.042

**Published:** 2026-06-01

**Authors:** Alexander A.W. Perkasa-Hendropriyono, David S. Perdanakusuma, Dwikora N. Utomo, Budiman Budiman, Sepehr S. Lajevardi, Gazi Hussain, Anand K. Deva

**Affiliations:** a Doctoral Program of Medical Science, Faculty of Medicine, Universitas Airlangga, Indonesia; b Department of Plastic Reconstructive and Aesthetic Surgery, Faculty of Medicine, Universitas Airlangga, Indonesia; c Department of Plastic Reconstructive and Aesthetic Surgery, Faculty of Medicine, Universitas Syiah Kuala, Indonesia; d Department of Plastic and Reconstructive Surgery, Faculty of Medicine, Health and Human Sciences, Macquarie University, Australia

**Keywords:** Exosome, Extracellular vesicle, Flap, Skin graft, Angiogenesis, Meta-Analysis

## Abstract

Flap necrosis and graft failure remain significant sources of morbidity in reconstructive and aesthetic surgery, yet no cell-free biological adjunct has been established to improve tissue survival after transfer. Exosomes are nano-sized extracellular vesicles capable of simultaneously modulating angiogenesis, inflammation, apoptosis, and oxidative stress, and have been investigated as candidate regenerative adjuncts in plastic surgery. This PROSPERO-registered systematic review and meta-analysis (CRD420251146650), conducted per PRISMA 2020, searched PubMed, OVID, Scopus, Web of Science, and Google Scholar through September 2025 and included 24 animal studies (19 flap; 5 skin graft). Flap and graft studies were analysed as separate populations. Random-effects meta-analysis suggested increased flap survival (*k* = 19; MD 35.54%; 95% CI 25.11–45.97; *p* < 0.0001) and angiogenesis (*k* = 19; SMD 3.60; 95% CI 2.66–4.54; *p* < 0.00001). However, heterogeneity was extreme (I² 76–97%), and funnel plot asymmetry with significant Egger's tests (both *p* < 0.001) indicated substantial small-study effects; the true effect is likely considerably smaller than these pooled estimates suggest, which should therefore be interpreted with caution. Perfusion, VEGF expression, and apoptosis generally favoured exosomes in narrative synthesis. In skin graft models, 4 of 5 studies reported improved graft take by days 10–14, but certainty was low. Allocation concealment was unreported in all studies and blinding unclear in most, representing a major methodological limitation that may further inflate observed effects. Preclinical evidence provides a biological rationale, but findings remain exploratory and hypothesis-generating. Standardised preclinical replication is required before clinical translation can be considered.

## Introduction

Exosomes, classified by the International Society for Extracellular Vesicles (ISEV) as small extracellular vesicles (sEV) smaller than 200 nm in diameter, are nano-sized intercellular messengers released by virtually all cell types.^1^, ^2^, ^3^ Enclosed by lipid bilayers and unable to self-replicate, exosomes are rich in proteins, cytokines, mRNAs, and microRNAs that can be transferred to recipient cells, enabling simultaneous modulation of multiple biological pathways.^1^^,^^2^^,^^4^, ^5^, ^6^ Unlike living stem cells, exosomes circumvent risks of immune rejection and tumorigenicity, and their lipid bilayer protects cargo from degradation in hostile wound microenvironments.^7^^,^^8^ The ability to lyophilize exosomes further confers practical advantages in storage and scalability over cell-based therapies.^7^, ^8^, ^9^, ^10^

Preclinical studies have demonstrated that exosomes exert regenerative effects through pathways directly relevant to tissue survival after surgical insult.^11^^,^^12^ These include promotion of angiogenesis via the HIF-1α/VEGFA axis, suppression of apoptosis through PI3K/AKT signaling, modulation of the inflammatory milieu by shifting macrophage polarisation from pro-inflammatory M1 to anti-inflammatory M2 phenotypes, and reduction of oxidative stress.^1^^,^^4^, ^5^, ^6^^,^^13^, ^14^, ^15^, ^16^, ^17^, ^18^ This multi-pathway capacity is theoretically advantageous in surgical settings where ischemic tissues face simultaneous hypoxia, inflammation, apoptosis, and oxidative damage. While exosomes have been explored across a broad range of applications including wound healing, fat grafting, and hair restoration, their potential to improve the survival of flaps and skin grafts has received comparatively limited systematic attention.^2^^,^^13^^,^^19^, ^20^, ^21^, ^22^, ^23^, ^24^, ^25^, ^26^

Flaps and skin grafts are the most fundamental tissue transfer techniques in plastic surgery and are used predominantly in reconstructive settings.^11^^,^^12^^,^^27^, ^28^, ^29^ Free and pedicled flaps are essential for reconstruction of complex defects following oncological resection, trauma, and burns, while skin grafts serve as workhorses for wound coverage in burn surgery, post-excisional defects, and chronic wounds.^11^^,^^12^^,^^27^, ^28^, ^29^ Both techniques also have important aesthetic applications: cervicofacial flaps in rhytidectomy, columellar flaps in rhinoplasty, and full-thickness skin grafts for lower eyelid reconstruction, free nipple grafts in reduction mammaplasty, and scar revision.^25^^,^^30^, ^31^, ^32^, ^33^, ^34^, ^35^

Despite their shared vulnerability to ischemia and necrosis, flaps and grafts rely on fundamentally distinct biological mechanisms for survival.^11^^,^^12^^,^^27^, ^28^, ^29^^,^^32^ Flaps maintain an intrinsic vascular pedicle and depend on adequate perfusion through existing vasculature; their survival is threatened when ischemia duration exceeds the tissue’s tolerance, triggering a cascade of hypoxia, inflammation, and cell death that progresses from distal to proximal zones.^11^^,^^12^^,^^27^, ^28^, ^29^^,^^32^ Skin grafts, by contrast, are completely devascularized tissues that must establish an entirely new blood supply from the recipient bed through the sequential processes of imbibition, inosculation, and revascularization. Graft failure occurs when this delicate process is disrupted by infection, hematoma, shear forces, or inadequate recipient bed vascularity.^11^^,^^12^^,^^27^, ^28^, ^29^^,^^32^ Despite these mechanistic differences, both flaps and grafts may be threatened by insufficient angiogenesis, excessive inflammation, and uncontrolled apoptosis, overlapping vulnerabilities that converge on a common endpoint of tissue loss.^11^^,^^12^^,^^27^, ^28^, ^29^^,^^32^ This shared pathobiology provides the rationale for evaluating exosome therapy across both tissue transfer types within a single systematic review, while maintaining analytical separation to respect their biological distinctions.

To date, no comprehensive synthesis has evaluated the preclinical evidence for exosome therapy in improving flap and skin graft survival. We therefore conducted a PROSPERO-registered systematic review and meta-analysis, following PRISMA 2020 guidelines, to quantify the effect of exosome therapy on flap survival and angiogenesis, synthesize evidence on skin graft outcomes, and identify which exosome parameters are associated with superior effects. Through this synthesis, we seek to provide a translational foundation for the design of future comparative clinical studies.

## Method

### Protocol registration

This PRISMA 2020-guided systematic review and meta-analysis was prospectively registered on PROSPERO (CRD420251146650). Reporting of animal studies was conducted in line with ARRIVE 2.0 “Essential 10.” MISEV 2018/2023 guidelines were used to classify and screen EVs.

### Search strategy

PubMed, OVID MEDLINE, Scopus, Web of Science Core Collection, and Google Scholar were searched from database inception to 12 September 2025, with searches executed between 12 and 15 September 2025. The search strategy combined controlled vocabulary and free-text terms related to exosomes or small extracellular vesicles, skin flaps or surgical flaps, skin grafts or skin transplantation, and animal or in vivo models. Database-specific syntax and indexing were used where applicable, including MeSH terms in PubMed and OVID MEDLINE. No language, date, or publication-type restrictions were applied. Google Scholar was searched as a supplementary source, with results screened by relevance. Reference lists of included studies and relevant reviews were also hand-searched for additional eligible records. The full database-specific search strings, search dates, and retrieval yields are provided in Supplementary Material 1.

### Eligibility criteria and outcome definitions

Eligibility was defined using the PICOS framework. Controlled in vivo animal studies evaluating exosome or small extracellular vesicle (sEV) therapy in skin flap or skin graft models were eligible if they included a placebo, vehicle, or untreated comparator and reported at least one efficacy outcome of interest. Exosomes or sEVs from any tissue or cellular source, isolation method, dose, route, timing, or carrier/scaffold were eligible, provided vesicle characterisation was sufficient for screening against contemporary MISEV criteria. We excluded in vitro or ex vivo studies, open-wound models without a flap or graft component, fat-graft models, studies without a control arm, human studies, and review articles, editorials, or letters. Vascularized composite allograft or other composite tissue transplant models were not classified as skin graft models because they represent a distinct vascularized reconstructive/transplant paradigm.

Because skin flaps and skin grafts have distinct survival mechanisms, eligible studies were categorized as flap models or skin graft models and synthesized separately; no pooled estimate combined the two model types. For flap studies, the primary clinical outcome was flap survival, usually expressed as viable flap area. For skin graft studies, the primary clinical outcome was graft take or graft survival, expressed as viable graft area or an equivalent postoperative viability endpoint. Mechanistic secondary outcomes of interest were angiogenesis/neovascularization, tissue perfusion, VEGF expression, and apoptosis, as these reflect pathways directly relevant to tissue survival after transfer.

Angiogenesis outcomes were defined as tissue-based measures of neovessel formation, including microvessel density, histologic vessel counts, CD31-positive endothelial staining, α-SMA-positive vessel staining, or comparable vascular immunohistochemical markers. Functional flow assessments (laser Doppler, laser speckle) were classified separately as perfusion outcomes. VEGF expression and apoptosis markers were also analysed separately and were not pooled with angiogenesis endpoints.

The analytical approach for each outcome was determined by the number of available studies rather than by outcome importance. Outcomes reported by ten or more studies (*k* ≥ 10) were eligible for meta-analysis; outcomes with fewer than ten studies were synthesized narratively to avoid imprecise between-study variance estimation and unreliable small-study effect testing. This threshold-based approach meant that flap survival (*k* = 19) and angiogenesis (*k* = 19) underwent meta-analysis, while perfusion (*k* = 7), VEGF (*k* = 7), apoptosis (*k* = 6), and skin graft take (*k* = 5) were synthesized narratively. The narrative treatment of skin graft take reflects the small number of eligible graft studies and the high clinical heterogeneity among them (mixed autologous and allogeneic models, variable outcome timepoints), not a lesser valuation of graft survival as an endpoint.

### Study selection

Three authors (A.A.W.P-H., B.B., D.S.P.) independently searched and screened articles. Titles and abstracts were screened by three reviewers (A.A.W.P-H., B.B., D.S.P.), with disagreements resolved by discussion. When consensus could not be reached, a fourth reviewer (D.N.U.) adjudicated. Full-text eligibility was assessed independently by A.A.W.P-H., B.B., D.S.P. with discrepancies resolved through discussion with the senior authors (S.S.L., G.H., A.K.D.). All inclusions and exclusions were made by human authors; Rayyan was used for deduplication and screening prioritisation only.

### Data extraction

Three authors recorded study data including publication details; animal model parameters (species, strain, sex, age, weight, flap/graft type and size, ischemia duration, follow-up timepoints); intervention (cellular source, tissue of origin, isolation method, dose, delivery route, timing, carrier or scaffold); study design (randomization, blinding, sample size per group); and outcomes (means, standard deviations, and sample sizes).

In instances where outcomes were only reported in graphs, digital graph extraction was performed. Study authors were contacted to confirm any missing information. Studies that reported in standard errors of the mean (SEM) were converted to standard deviation (SD) by SD = SEM × √n.

### Risk of bias and study quality

All studies were assessed with SYRCLE Risk of Bias. Domains included sequence generation, baseline similarity, allocation concealment, random housing, blinding of caregivers/investigators, random outcome assessment, blinding of outcome assessment, incomplete outcome data, selective reporting, and other bias. Each domain was graded low, unclear, or high risk. ARRIVE 2.0 Essential 10 items and MISEV 2018/2023 compliance were also assessed.

### Statistical analysis

Flap and skin graft studies were analysed as separate populations throughout. Data were analysed through random-effects models (REML). For flap survival (*k* = 19), mean difference (MD) in percentage survival area was calculated. For angiogenesis (*k* = 19), standardised mean difference (SMD) was used because studies employed different histological measures (microvessel density, CD31+ vessel counts, α-SMA+ vessel counts) that share the same construct but differ in units and scale. The use of SMD to pool these heterogeneous measures is acknowledged as a limitation, as small within-study variance in tightly controlled animal experiments can yield large effect sizes. Heterogeneity was assessed using τ² (REML), Cochran’s Q, and I² with *p* < 0.10 considered significant. Effect estimates were reported with 95% confidence intervals. Small-study effects were assessed by funnel plots and Egger’s regression for endpoints with *k* ≥ 10.

Narrative synthesis was performed for perfusion (*k* = 7), VEGF (*k* = 7), apoptosis (*k* = 6), and skin graft take (*k* = 5), summarized in Table 6. Subgroup analyses explored exosome cellular source (adipose-derived vs bone marrow-derived) and species origin (human vs animal-derived) within the flap meta-analysis. Meta-regression was not performed as subgroup sizes were insufficient. Sensitivity analysis included leave-one-out analyses. Statistical analysis was performed using Review Manager (version 5.4) and Jamovi (version 2.7.6; meta and metafor packages). Values of *p* < 0.05 were considered significant (except heterogeneity at *p* < 0.10).

## Results

**Fig. 1:**
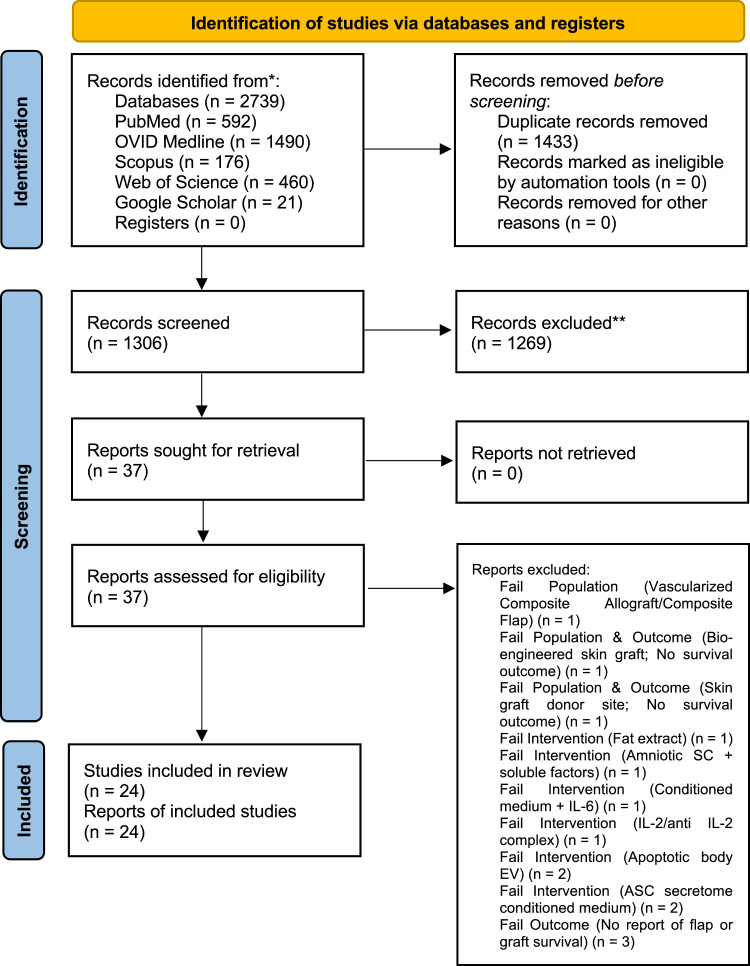
PRISMA 2020 flow diagram.

The search yielded 2739 records across all databases. After removal of duplicates, 1306 records underwent title and abstract screening. Thirty-seven articles were selected for full-text assessment. Twenty-four studies (19 flap and 5 skin graft) met the inclusion criteria and were included for further quantitative analysis (Fig. 1).

The included studies were published between 2017 and 2025. Thirteen studies used mouse models, 10 studies used rat models, and one study used miniature pigs. The sources of exosomes were varied, with adipose-derived stem cells (ADSCs) (*n* = 9) and bone marrow-derived mesenchymal stem cells (BMSCs) (*n* = 7) being the most common. Exosome characterisation methods and dosing regimens varied considerably. Administration routes included subcutaneous injection (*n* = 11), intravenous (*n* = 7), intradermal (*n* = 4), and hydrogel scaffolds under flaps (*n* = 2). Study characteristics for flaps are presented in Table 1 and for skin grafts in Table 2. Other characteristics provided in Supplement 2.

**Table 1:** Systematic review of flap studies.

Study	Animal Model (Species; Strain; Sex; n)	Flap Type (Size; Location; Ischemia)	Exosome Source	Dose; Route; Timing; Frequency	Flap Survival (%) Exosome vs. Control	Angiogenesis (vessels/field) Exosome vs. Control	SYRCLE RoB (Low / Unclear)
**Bai et al., 201,8**^36^	Rat; SD; M; 260–320 g; *n* = 6	SIEA flap (6 × 9 cm); abdomen; 6 h ischemia	Human H₂O₂-ADSC Exo	100 µg; SC injection; Post-op; Single	**Exo:** 73.977 ± 10.027 **Ctrl:** 5.263 ± 5.730	**Exo:** 35.974 ± 18.451 **Ctrl:** 1.429 ± 4.454	6 Low / 4 Unclear
**Chang et al., 202,5**^37^	Mouse; C57BL/6; M; 8 wk; *n* = 3	Skin flap (2 × 4 cm); back; random pattern	Human HemSC Exo	32 µg; Hydrogel scaffold; Intra-op; Single	**Exo:** 85.124 ± 9.917 **Ctrl:** 11.157 ± 5.785	**Exo:** 672.955 ± 94.091 **Ctrl:** 94.091 ± 53.182	6 Low / 4 Unclear
**Deng et al., 20,23**^4^	Rat; SD; M; 12 wk; 350–400 g; *n* = 5	CSEA flap (3 × 6 cm); abdomen; 6 h ischemia	Rat BMSC Hypoxia-PC Exo	200 µg; SC injection; Daily × 7 days	**Exo:** 68.644 ± 37.853 **Ctrl:** 11.299 ± 5.650	**Exo:** 169.847 ± 54.707 **Ctrl:** 44.529 ± 21.628	7 Low / 3 Unclear
**Ding et al., 202,4**^38^	Rat; Wistar; M; 4–6 wk; *n* = 3	Trans-territory perforator flap (10 × 2.5 cm); dorsal; 2 choke zones	Human BMSC Hypoxia-PT Exo	20 µg; SC into choke zone II; Post-op; Single	**Exo:** 79.699 ± 6.867 **Ctrl:** 63.614 ± 8.133	**Exo:** 31.670 ± 3.050 **Ctrl:** 16.000 ± 3.600	6 Low / 4 Unclear
**Ge et al., 202,3**^39^	Mouse; C57BL/6; M; 8–10 wk; 20–25 g; *n* = 6	Diabetic skin flap (1.5 × 4.5 cm); back; random pattern	Human ADSC miR-132-OE Exo	100 µg; SC injection; Post-op; Single	**Exo:** 87.963 ± 7.407 **Ctrl:** 41.667 ± 18.981	**Exo:** 59.645 ± 7.258 **Ctrl:** 11.519 ± 5.996	6 Low / 4 Unclear
**Guo et al., 202,2**^40^	Rat; SD; M; 8 wk; 400–450 g; *n* = 5	Skin flap (3 × 12 cm); back; random pattern	Human H₂O₂-HUVEC Exo	500 µg; SC injection; Post-op; Single	**Exo:** 79.300 ± 9.276 **Ctrl:** 51.000 ± 6.808	**Exo:** 62.295 ± 27.459 **Ctrl:** 12.705 ± 12.295	6 Low / 4 Unclear
**Liu et al., 202,4**^41^	Mouse; C57BL/6; M; *n* = 6	Skin flap (1 × 3 cm); dorsal; random pattern	Human ADSC Exo + Curcumin	32 µg; Hydrogel scaffold; Intra-op; Single	**Exo:** 71.830 ± 9.881 **Ctrl:** 41.430 ± 10.095	**Exo:** 4.409 ± 0.745 **Ctrl:** 0.964 ± 0.803	7 Low / 3 Unclear
**Liu et al., 20,25**^5^	Mouse; BALB/c-nude; M; *n* = 8	Skin flap (1.5 × 4.5 cm); dorsal; random; 6 h ischemia	Human Platelet sEV	10 µg; SC injection; Every 2 days; Multiple	**Exo:** 80.089 ± 6.578 **Ctrl:** 30.311 ± 8.000	**Exo:** 3.627 ± 0.229 **Ctrl:** 0.966 ± 0.186	6 Low / 4 Unclear
**Luo et al., 20,24**^6^	Mouse; BALB/c; M; 8 wk; 22–25 g; *n* = 6	Fasciocutaneous flap (5.5 × 1.5 cm); dorsal; random pattern	Human M2 Macrophage-Derived Exo	500 µg; IV injection; Post-op; Single	**Exo:** 86.170 ± 4.660 **Ctrl:** 47.670 ± 9.800	**Exo:** 19.500 ± 3.500 **Ctrl:** 4.160 ± 2.130	6 Low / 4 Unclear
**Mayo et al., 201,9**^42^	Mouse; BALB/c; M; 9–12 wk; *n* = 5	Skin flap (4 × 2 cm); back; random pattern	Human H₂O₂-ADSC EVs	3 × 10¹⁰ particles; ID injection; Post-op; Single	**Exo:** 48.947 ± 11.180 **Ctrl:** 32.237 ± 8.238	**Exo:** 6.790 ± 1.789 **Ctrl:** 2.769 ± 0.414	7 Low / 3 Unclear
**Ngo et al., 202,2**^43^	Mouse; C57BL/6 J; F; 8 wk; *n* = 9	Skin flap (3 × 2 cm); dorsum; permanent ischemia	Human UCB-EPC Exo	60 µg; ID injection; Post-op; Single	**Exo:** 74.571 ± 14.286 **Ctrl:** 31.429 ± 18.286	**Exo:** 96.204 ± 24.476 **Ctrl:** 31.615 ± 6.799	6 Low / 4 Unclear
**Niu et al., 202,2**^44^	Rat; SD; M; 6–8 wk; 250–300 g; *n* = 6	Abdominal free flap (3 × 5 cm); epigastric vessel; 4 h ischemia	Rat BMSC Exo	500 µg; IV tail vein; Day 0.1.3.5; 4 doses	**Exo:** 93.920 ± 3.878 **Ctrl:** 69.392 ± 12.369	**Exo:** 41.250 ± 12.115 **Ctrl:** 12.837 ± 6.635	6 Low / 4 Unclear
**Pu et al., 201,7**^45^	Mouse; C57BL/6 J; M; 25–30 g; *n* = 6	Extended pectoral flap (4 × 1 cm); 3 h ischemia	Human ADSC Exo	100 µg/mL; SC injection; Post-op; Single	**Exo:** 72.421 ± 31.591 **Ctrl:** 35.714 ± 4.374	**Exo:** 13.802 ± 2.771 **Ctrl:** 5.754 ± 3.484	8 Low / 2 Unclear
**Shi et al., 202,3**^46^	Rat; SD; M; 250–300 g; *n* = 12	SIEA flap (3 × 6 cm); abdomen; 6 h ischemia	Human hDPSC Exo	100 µg; SC injection; Post-op; Single	**Exo:** 79.446 ± 17.552 **Ctrl:** 39.261 ± 15.704	**Exo:** 26.950 ± 10.969 **Ctrl:** 12.293 ± 3.404	6 Low / 4 Unclear
**Sun et al., 202,5**^47^	Rat; SD; M; 4 wk; *n* = 4	Free flap (multi-territory perforator) (11 × 2.5 cm); thoracodorsal a.	Rat BMSC Exo + Metformin	1 × 10⁵; IV (thoracodorsal a.); Post-op; Single	**Exo:** 100.000 ± 1.000 **Ctrl:** 89.131 ± 10.696	**Exo:** 21.899 ± 6.076 **Ctrl:** 6.519 ± 3.165	8 Low / 2 Unclear
**Wu et al., 202,2**^48^	Rat; SD; M; *n* = 10	Skin flap (9 × 3 cm); back; random pattern	Human Hypoxia-ADSC EVs	10 µg; ID injection; Post-op; Single	**Exo:** 70.900 ± 2.800 **Ctrl:** 21.400 ± 2.700	**Exo:** 83.600 ± 2.500 **Ctrl:** 48.700 ± 2.000	8 Low / 2 Unclear
**Xie et al., 201,9**^49^	Rat; SD; M; 216 ± 13 g; *n* = 15	Skin flap (9 × 3 cm); back; random pattern	Rat BMSC Exo	135 µg; ID injection; Post-op; Single	**Exo:** 56.900 ± 4.400 **Ctrl:** 49.500 ± 3.100	**Exo:** 36.400 ± 7.400 **Ctrl:** 30.500 ± 5.400	6 Low / 4 Unclear
**Zhang et al., 202,4**^50^	Mouse; C57BL/6; M; 8–10 wk; 25–30 g; *n* = 5	Skin flap (1.5 × 4.5 cm); dorsal; random; permanent ischemia	Human FGF1-PC ADSC Exo	100 µg; SC injection; Post-op; Single	**Exo:** 73.970 ± 5.336 **Ctrl:** 45.553 ± 5.821	**Exo:** 92.371 ± 8.225 **Ctrl:** 29.428 ± 7.311	8 Low / 2 Unclear
**Zhu et al., 202,5**^51^	Rat; SD; M; 8 wk; 280–320 g; *n* = 6	Skin flap (9 × 3 cm); back; random pattern	Rat BMSC Exo	200 µg; IV tail vein; Post-op; Single	**Exo:** 74.780 ± 7.900 **Ctrl:** 56.370 ± 3.200	**Exo:** 37.500 ± 5.250 **Ctrl:** 21.900 ± 3.150	6 Low / 4 Unclear

**Abbreviations:** SD = Sprague-Dawley; M = Male; F = Female; wk = weeks; SIEA = superficial inferior epigastric artery; CSEA = circumflex superficial epigastric artery; ADSC = adipose-derived stem cell; BMSC = bone marrow-derived mesenchymal stem cell; HemSC = hemangioma stem cell; hDPSC = human dental pulp stem cell; UCB-EPC = umbilical cord blood endothelial progenitor cell; HUVEC = human umbilical vein endothelial cell; PC = preconditioned; PT = pretreated; OE = overexpressing; Exo = exosome(s); EV(s) = extracellular vesicle(s); sEV = small extracellular vesicle; SC = subcutaneous; IV = intravenous; ID = intradermal; RoB = risk of bias.

**Note:** SYRCLE RoB is reported as domain-level counts (e.g., “6 Low / 4 Unclear” = 6 domains rated Low risk. 4 domains rated Unclear risk) rather than an overall judgment. as SYRCLE does not prescribe a formal algorithm for overall risk classification. No study received a High risk rating in any domain. Detailed domain-level assessments are provided in Table 3. Detailed isolation protocols. characterization markers. and secondary outcome values are provided in Supplementary Table 2. Values are reported as mean ± SD.

**Table 2:** Systematic review of skin graft studies.

Study	Animal Model (Species; Strain; Sex; n)	Flap Type (Size; Location; Ischemia)	Exosome Source	Dose; Route; Timing; Frequency	Flap Survival (%) Exosome vs. Control	Angiogenesis (vessels/field) Exosome vs. Control	SYRCLE RoB (Low / Unclear)
**Bai et al., 201,8**^36^	Rat; SD; M; 260–320 g; *n* = 6	SIEA flap (6 × 9 cm); abdomen; 6 h ischemia	Human H₂O₂-ADSC Exo	100 µg; SC injection; Post-op; Single	**Exo:** 73.977 ± 10.027 **Ctrl:** 5.263 ± 5.730	**Exo:** 35.974 ± 18.451 **Ctrl:** 1.429 ± 4.454	6 Low / 4 Unclear
**Chang et al., 202,5**^37^	Mouse; C57BL/6; M; 8 wk; *n* = 3	Skin flap (2 × 4 cm); back; random pattern	Human HemSC Exo	32 µg; Hydrogel scaffold; Intra-op; Single	**Exo:** 85.124 ± 9.917 **Ctrl:** 11.157 ± 5.785	**Exo:** 672.955 ± 94.091 **Ctrl:** 94.091 ± 53.182	6 Low / 4 Unclear
**Deng et al., 20,23**^4^	Rat; SD; M; 12 wk; 350–400 g; *n* = 5	CSEA flap (3 × 6 cm); abdomen; 6 h ischemia	Rat BMSC Hypoxia-PC Exo	200 µg; SC injection; Daily × 7 days	**Exo:** 68.644 ± 37.853 **Ctrl:** 11.299 ± 5.650	**Exo:** 169.847 ± 54.707 **Ctrl:** 44.529 ± 21.628	7 Low / 3 Unclear
**Ding et al., 202,4**^38^	Rat; Wistar; M; 4–6 wk; *n* = 3	Trans-territory perforator flap (10 × 2.5 cm); dorsal; 2 choke zones	Human BMSC Hypoxia-PT Exo	20 µg; SC into choke zone II; Post-op; Single	**Exo:** 79.699 ± 6.867 **Ctrl:** 63.614 ± 8.133	**Exo:** 31.670 ± 3.050 **Ctrl:** 16.000 ± 3.600	6 Low / 4 Unclear
**Ge et al., 202,3**^39^	Mouse; C57BL/6; M; 8–10 wk; 20–25 g; *n* = 6	Diabetic skin flap (1.5 × 4.5 cm); back; random pattern	Human ADSC miR-132-OE Exo	100 µg; SC injection; Post-op; Single	**Exo:** 87.963 ± 7.407 **Ctrl:** 41.667 ± 18.981	**Exo:** 59.645 ± 7.258 **Ctrl:** 11.519 ± 5.996	6 Low / 4 Unclear
**Guo et al., 202,2**^40^	Rat; SD; M; 8 wk; 400–450 g; *n* = 5	Skin flap (3 × 12 cm); back; random pattern	Human H₂O₂-HUVEC Exo	500 µg; SC injection; Post-op; Single	**Exo:** 79.300 ± 9.276 **Ctrl:** 51.000 ± 6.808	**Exo:** 62.295 ± 27.459 **Ctrl:** 12.705 ± 12.295	6 Low / 4 Unclear
**Liu et al., 202,4**^41^	Mouse; C57BL/6; M; *n* = 6	Skin flap (1 × 3 cm); dorsal; random pattern	Human ADSC Exo + Curcumin	32 µg; Hydrogel scaffold; Intra-op; Single	**Exo:** 71.830 ± 9.881 **Ctrl:** 41.430 ± 10.095	**Exo:** 4.409 ± 0.745 **Ctrl:** 0.964 ± 0.803	7 Low / 3 Unclear
**Liu et al., 20,25**^5^	Mouse; BALB/c-nude; M; *n* = 8	Skin flap (1.5 × 4.5 cm); dorsal; random; 6 h ischemia	Human Platelet sEV	10 µg; SC injection; Every 2 days; Multiple	**Exo:** 80.089 ± 6.578 **Ctrl:** 30.311 ± 8.000	**Exo:** 3.627 ± 0.229 **Ctrl:** 0.966 ± 0.186	6 Low / 4 Unclear
**Luo et al., 20,24**^6^	Mouse; BALB/c; M; 8 wk; 22–25 g; *n* = 6	Fasciocutaneous flap (5.5 × 1.5 cm); dorsal; random pattern	Human M2 Macrophage-Derived Exo	500 µg; IV injection; Post-op; Single	**Exo:** 86.170 ± 4.660 **Ctrl:** 47.670 ± 9.800	**Exo:** 19.500 ± 3.500 **Ctrl:** 4.160 ± 2.130	6 Low / 4 Unclear
**Mayo et al., 201,9**^42^	Mouse; BALB/c; M; 9–12 wk; *n* = 5	Skin flap (4 × 2 cm); back; random pattern	Human H₂O₂-ADSC EVs	3 × 10¹⁰ particles; ID injection; Post-op; Single	**Exo:** 48.947 ± 11.180 **Ctrl:** 32.237 ± 8.238	**Exo:** 6.790 ± 1.789 **Ctrl:** 2.769 ± 0.414	7 Low / 3 Unclear
**Ngo et al., 202,2**^43^	Mouse; C57BL/6 J; F; 8 wk; *n* = 9	Skin flap (3 × 2 cm); dorsum; permanent ischemia	Human UCB-EPC Exo	60 µg; ID injection; Post-op; Single	**Exo:** 74.571 ± 14.286 **Ctrl:** 31.429 ± 18.286	**Exo:** 96.204 ± 24.476 **Ctrl:** 31.615 ± 6.799	6 Low / 4 Unclear
**Niu et al., 202,2**^44^	Rat; SD; M; 6–8 wk; 250–300 g; *n* = 6	Abdominal free flap (3 × 5 cm); epigastric vessel; 4 h ischemia	Rat BMSC Exo	500 µg; IV tail vein; Day 0.1.3.5; 4 doses	**Exo:** 93.920 ± 3.878 **Ctrl:** 69.392 ± 12.369	**Exo:** 41.250 ± 12.115 **Ctrl:** 12.837 ± 6.635	6 Low / 4 Unclear
**Pu et al., 201,7**^45^	Mouse; C57BL/6 J; M; 25–30 g; *n* = 6	Extended pectoral flap (4 × 1 cm); 3 h ischemia	Human ADSC Exo	100 µg/mL; SC injection; Post-op; Single	**Exo:** 72.421 ± 31.591 **Ctrl:** 35.714 ± 4.374	**Exo:** 13.802 ± 2.771 **Ctrl:** 5.754 ± 3.484	8 Low / 2 Unclear
**Shi et al., 202,3**^46^	Rat; SD; M; 250–300 g; *n* = 12	SIEA flap (3 × 6 cm); abdomen; 6 h ischemia	Human hDPSC Exo	100 µg; SC injection; Post-op; Single	**Exo:** 79.446 ± 17.552 **Ctrl:** 39.261 ± 15.704	**Exo:** 26.950 ± 10.969 **Ctrl:** 12.293 ± 3.404	6 Low / 4 Unclear
**Sun et al., 202,5**^47^	Rat; SD; M; 4 wk; *n* = 4	Free flap (multi-territory perforator) (11 × 2.5 cm); thoracodorsal a.	Rat BMSC Exo + Metformin	1 × 10⁵; IV (thoracodorsal a.); Post-op; Single	**Exo:** 100.000 ± 1.000 **Ctrl:** 89.131 ± 10.696	**Exo:** 21.899 ± 6.076 **Ctrl:** 6.519 ± 3.165	8 Low / 2 Unclear
**Wu et al., 202,2**^48^	Rat; SD; M; *n* = 10	Skin flap (9 × 3 cm); back; random pattern	Human Hypoxia-ADSC EVs	10 µg; ID injection; Post-op; Single	**Exo:** 70.900 ± 2.800 **Ctrl:** 21.400 ± 2.700	**Exo:** 83.600 ± 2.500 **Ctrl:** 48.700 ± 2.000	8 Low / 2 Unclear
**Xie et al., 201,9**^49^	Rat; SD; M; 216 ± 13 g; *n* = 15	Skin flap (9 × 3 cm); back; random pattern	Rat BMSC Exo	135 µg; ID injection; Post-op; Single	**Exo:** 56.900 ± 4.400 **Ctrl:** 49.500 ± 3.100	**Exo:** 36.400 ± 7.400 **Ctrl:** 30.500 ± 5.400	6 Low / 4 Unclear
**Zhang et al., 202,4**^50^	Mouse; C57BL/6; M; 8–10 wk; 25–30 g; *n* = 5	Skin flap (1.5 × 4.5 cm); dorsal; random; permanent ischemia	Human FGF1-PC ADSC Exo	100 µg; SC injection; Post-op; Single	**Exo:** 73.970 ± 5.336 **Ctrl:** 45.553 ± 5.821	**Exo:** 92.371 ± 8.225 **Ctrl:** 29.428 ± 7.311	8 Low / 2 Unclear
**Zhu et al., 202,5**^51^	Rat; SD; M; 8 wk; 280–320 g; *n* = 6	Skin flap (9 × 3 cm); back; random pattern	Rat BMSC Exo	200 µg; IV tail vein; Post-op; Single	**Exo:** 74.780 ± 7.900 **Ctrl:** 56.370 ± 3.200	**Exo:** 37.500 ± 5.250 **Ctrl:** 21.900 ± 3.150	6 Low / 4 Unclear

**Abbreviations:** SD = Sprague-Dawley; M = Male; F = Female; wk = weeks; SIEA = superficial inferior epigastric artery; CSEA = circumflex superficial epigastric artery; ADSC = adipose-derived stem cell; BMSC = bone marrow-derived mesenchymal stem cell; HemSC = hemangioma stem cell; hDPSC = human dental pulp stem cell; UCB-EPC = umbilical cord blood endothelial progenitor cell; HUVEC = human umbilical vein endothelial cell; PC = preconditioned; PT = pretreated; OE = overexpressing; Exo = exosome(s); EV(s) = extracellular vesicle(s); sEV = small extracellular vesicle; SC = subcutaneous; IV = intravenous; ID = intradermal; RoB = risk of bias.

**Note:** SYRCLE RoB is reported as domain-level counts (e.g., “6 Low / 4 Unclear” = 6 domains rated Low risk. 4 domains rated Unclear risk) rather than an overall judgment. as SYRCLE does not prescribe a formal algorithm for overall risk classification. No study received a High risk rating in any domain. Detailed domain-level assessments are provided in Table 3. Detailed isolation protocols. characterization markers. and secondary outcome values are provided in Supplementary Table 2. Values are reported as mean ± SD.

No study was rated high risk in any SYRCLE domain. However, allocation concealment was unclear in all 24 studies (100%), random housing in 20 (83%), caregiver/investigator blinding in 18 (75%), and outcome assessor blinding in 18 (75%). Sequence generation, baseline characteristics, random outcome assessment, incomplete outcome data, selective reporting, and other sources of bias were predominantly rated low risk (Table 3). The lack of reporting on concealment and blinding limits confidence in the overall evidence base.

**Table 3:** SYRCLE risk of bias assessment.

Study	1. Sequence Generation (Selection)	2. Baseline Characteristics (Selection)	3. Allocation Concealment (Selection)	4. Random Housing (Performance)	5. Caregiver/ Investigator Blinding (Performance)	6. Random Outcome Assessment (Detection)	7. Outcome Assessor Blinding (Detection)	8. Incomplete Outcome Data (Attrition)	9. Selective Outcome Reporting (Reporting)	10. Other Sources of Bias
**Bai et al., 2018**	Low	Low	Unclear	Unclear	Unclear	Low	Unclear	Low	Low	Low
**Campos-Mora et al., 2021**	Low	Low	Unclear	Low	Unclear	Low	Unclear	Low	Low	Low
**Chang et al., 2025**	Low	Low	Unclear	Unclear	Unclear	Low	Unclear	Low	Low	Low
**Deng et al., 2023**	Low	Low	Unclear	Low	Unclear	Low	Unclear	Low	Low	Low
**Ding et al., 2024**	Low	Low	Unclear	Unclear	Unclear	Low	Unclear	Low	Low	Low
**Ge et al., 2023**	Low	Low	Unclear	Unclear	Unclear	Low	Unclear	Low	Low	Low
**Guo et al., 2022**	Low	Low	Unclear	Unclear	Unclear	Low	Unclear	Low	Low	Low
**Li et al., 2019**	Unclear	Low	Unclear	Unclear	Unclear	Low	Unclear	Low	Low	Low
**Pujun Li et al., 2025**	Low	Low	Unclear	Low	Unclear	Low	Unclear	Low	Low	Low
**Xinqiang Li et al., 2025**	Unclear	Low	Unclear	Unclear	Unclear	Low	Unclear	Low	Low	Low
**Liu et al., 2024**	Low	Low	Unclear	Low	Unclear	Low	Unclear	Low	Low	Low
**Liu et al., 2025**	Low	Low	Unclear	Unclear	Unclear	Low	Unclear	Low	Low	Low
**Luo et al., 2024**	Low	Low	Unclear	Unclear	Unclear	Low	Unclear	Low	Low	Low
**Mayo et al., 2019**	Unclear	Low	Unclear	Unclear	Low	Low	Low	Low	Low	Low
**Ngo et al., 2022**	Low	Low	Unclear	Unclear	Unclear	Low	Unclear	Low	Low	Low
**Niu et al., 2022**	Low	Low	Unclear	Unclear	Unclear	Low	Unclear	Low	Low	Low
**Pu et al., 2017**	Low	Low	Unclear	Unclear	Low	Low	Low	Low	Low	Low
**Shi et al., 2023**	Low	Low	Unclear	Unclear	Unclear	Low	Unclear	Low	Low	Low
**Sun et al., 2025**	Low	Low	Unclear	Unclear	Low	Low	Low	Low	Low	Low
**Wu et al., 2022**	Low	Low	Unclear	Unclear	Low	Low	Low	Low	Low	Low
**Xie et al., 2019**	Low	Low	Unclear	Unclear	Unclear	Low	Unclear	Low	Low	Low
**Zhang et al., 2023**	Low	Low	Unclear	Unclear	Low	Low	Low	Low	Low	Low
**Zhang et al., 2024**	Low	Low	Unclear	Unclear	Low	Low	Low	Low	Low	Low
**Zhu et al., 2025**	Low	Low	Unclear	Unclear	Unclear	Low	Unclear	Low	Low	Low
**Summary (n/24)**	**21L** **3U**	**24L** **0U**	**0L** **24U**	**4L** **20U**	**6L** **18U**	**24L** **0U**	**6L** **18U**	**24L** **0U**	**24L** **0U**	**24L** **0U**

**Color coding:** Green = Low risk; Amber = Unclear risk; Red = High risk (none identified). **Abbreviations:** L = Low risk; U = Unclear risk. SYRCLE = SYstematic Review Centre for Laboratory animal Experimentation.

**Note:** No overall risk of bias judgment is provided, as SYRCLE does not prescribe a formal algorithm for deriving an overall score. Domain-level judgments are presented to assess the methodological limitations of each study. Allocation concealment was unclear in all 24 studies (100%), reflecting a systemic reporting gap in preclinical exosome research. Unclear risk indicates insufficient reporting to determine whether the methodological safeguard was implemented; it should not be interpreted as low risk.

## Primary endpoints (Meta-Analysis)

### Flap survival

**Fig. 2:**
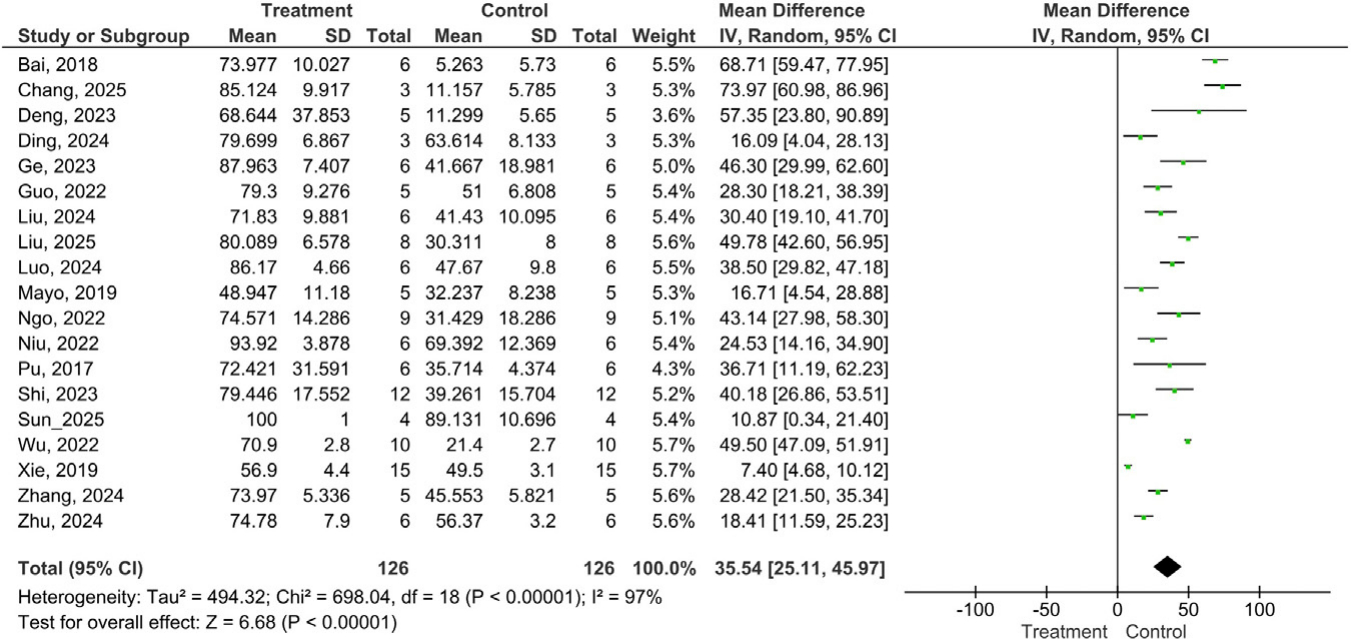
Meta-analysis of flap survival.

**Fig. 3:**
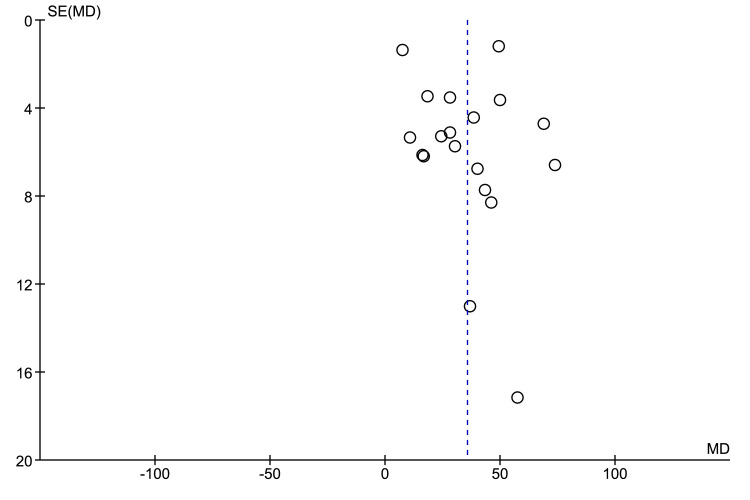
Funnel plot of flap survival.

Meta-analysis with random effects of 19 studies demonstrated a significant positive effect of exosomes on improving flap survival (Table 1 and Fig. 2). Mean difference (MD) was 35.54% (95% CI 25.11–45.97; Z = 6.68; *p* < 0.0001). Heterogeneity was substantial (I² = 97%; τ² = 494.32; χ² = 698.04; *p* < 0.0001). Egger’s regression (6.735; *p* < 0.001) and funnel plot asymmetry suggest potential small-study effects (Fig. 3 and Table 4). Individual study effects ranged from 7.40% to 73.97%. Studies with the smallest sample sizes exhibited the widest confidence intervals, reflecting the imprecision inherent in small-sample preclinical experiments. The study by Sun et al. (2025) reported the largest effect, although with a notably narrow CI that may reflect low within-study variance. Leave-one-out analysis provided in Supplement 3.

**Table 4:** Egger’s test for flap survival.

Publication Bias Assessment		
Test Name	value	P
Fail-Safe N	1654.000	<0.001
Begg and Mazumdar Rank Correlation	0.684	<0.001
Egger's Regression	6.735	<0.001
Trim and Fill Number of Studies	0.000	.

Note. Fail-safe N Calculation Using the Rosenthal Approach.

### Angiogenesis/Neovascularization

**Fig. 4:**
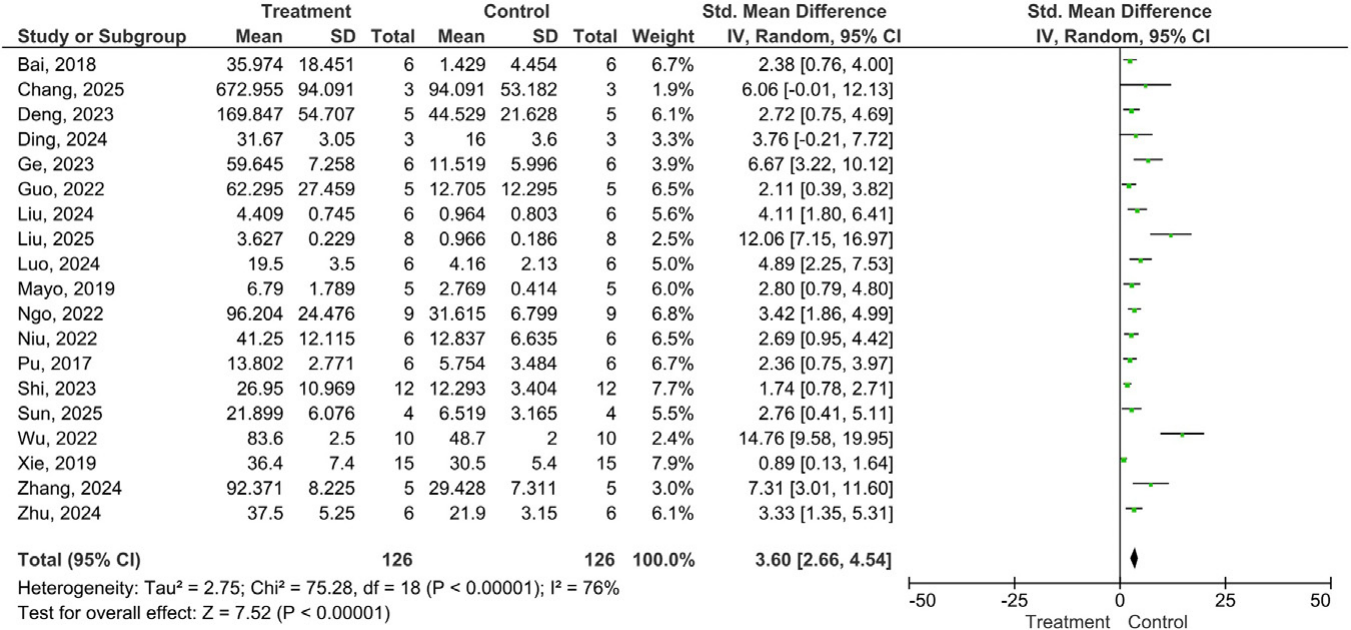
Meta-analysis of angiogenesis/neovascularization.

**Fig. 5:**
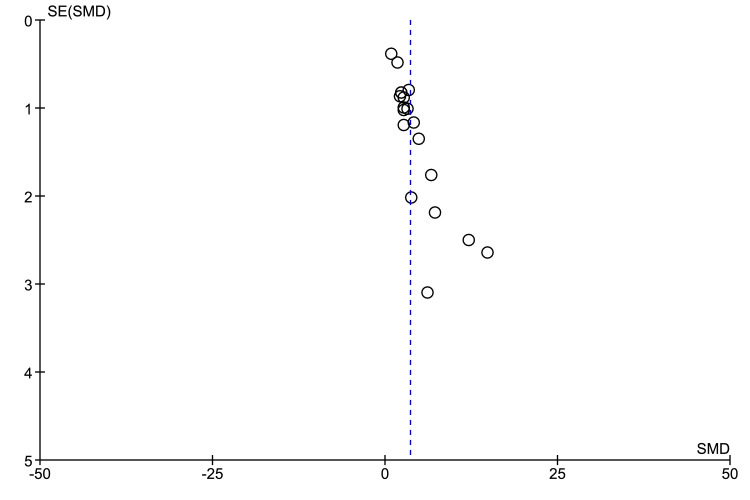
Funnel plot of angiogenesis/neovascularization.

Nineteen studies reported outcomes of angiogenesis or neovascularization. Markers included microvessel density (MVD) and relative neovascularization scores. Exosomes were associated with greater angiogenesis than control (SMD = 3.60; 95% CI 2.66–4.54; Z = 7.52; *p* < 0.00001) (Table 1 and Fig. 4). Heterogeneity was substantial (I^2^ = 76%, τ² = 2.75, χ^2^ = 75.28, and *p* < 0.00001). Funnel-plot asymmetry and Egger’s regression (9.088, *p* < 0.001) suggested small-study effects (Fig. 5; Table 5). Wu et al. (2022) was an outlier with an SMD of 14.76, largely driven by exceptionally low within-study variance; excluding this study did not materially alter the pooled estimate on sensitivity analysis (Supplement 4). Two studies had confidence intervals crossing zero, consistent with their small sample sizes.

**Table 5:** Egger’s test for angiogenesis/neovascularization.

Publication Bias Assessment		
Test Name	value	p
Fail-Safe N	1805.000	<0.001
Begg and Mazumdar Rank Correlation	0.766	<0.001
Egger's Regression	9.088	<0.001
Trim and Fill Number of Studies	0.000	.

Note. Fail-safe N Calculation Using the Rosenthal Approach.

### Exploratory subgroups (Exosome source and species)

Sub-group analysis suggested that ADSC-exos provided a more significant effect on flap survival than BMSC-exos. Trends also favoured human exosomes over animal exosomes (Table 1). Subgroup sizes were quite small, therefore these observations should be considered hypothesis-generating only.

### Secondary endpoints (Narrative synthesis)

Perfusion (*k* = 7), VEGF (*k* = 7), apoptosis (*k* = 6), and skin graft take (*k* = 5) were synthesized narratively because each was reported by fewer than 10 studies. Table 6 presents a structured summary of these four outcomes, reporting for each the number of contributing studies, direction of effect, consistency across studies, effect range, key mechanistic observations, and an informal certainty rating.

**Table 6:** Summary of Secondary Outcomes (Narrative Synthesis) Summary of Secondary Outcomes.

Outcome	k	Direction of Effect	Consistency	Effect Range	Key Observations	Certainty of Evidence
**Flap Perfusion***Laser Doppler (4); ICG angiography (3)*	7	**↑ Favors exosomes**	**6/7** studies reported statistically significant improvement (*p* < 0.05)	Individual study SMD 1.86–5.23 (moderate-to-large effects)	• Perfusion findings directionally consistent with angiogenesis meta-analysis (SMD 3.60) • 1 study (Zhu 2024) showed non-significant trend favoring exosomes (*p* > 0.05) • Measurement heterogeneity (Doppler vs. ICG) precluded meta-analysis	**Low–Moderate***Small k; heterogeneous measurement methods; no pooled estimate calculated*
**VEGF Expression***IHC (4); ELISA (2); Western blot (3)*	7	**↑ Upregulated**	**7/7** studies showed significant VEGF upregulation	IHC: 1.5–2.8 × fold increase (VEGF+ cells/HPF) Protein: 1.00–2.26 × fold increase vs. control	• Dose-dependent trend: doses > 100 µg yielded greater VEGF upregulation • HIF-1α/VEGFA axis implicated in 5 studies (Luo, Wu, Deng, Guo, Ngo) • Hypoxia-preconditioned exosomes showed enhanced VEGF induction vs. normoxic • Some studies used > 1 assay method; all methods showed consistent direction	**Low–Moderate***Consistent direction but variable assay methods; small k limits precision*
**Apoptosis***TUNEL (4); Caspase-3 IHC (3)*	6	**↓ Reduced**	**6/6** studies reported significant reduction in apoptotic index (*p* < 0.05)	Consistent significant reduction across all studies; quantitative pooling not performed	• PI3K/AKT anti-apoptotic activation (4 studies) • Caspase-3 suppression (5 studies); Bcl-2 upregulation (3 studies) • Anti-apoptotic effect most pronounced at distal ischemic flap zones • Direction inversely consistent with improved flap survival (as expected biologically)	**Low–Moderate***Small k; consistent direction; unclear blinding in majority of studies*
**Skin Graft Take***Macroscopic area survival (%); rodent (5), pig (1)*	5	**↑ Favors exosomes***(time-dependent)*	**Day 7:** 2/5 significant **Day 10–14:** 4/5 significant	Day 14: exosome 78–92% vs. control 38–55% 1 study non-significant (72%vs. 60%)	• Limited separation at day 7 likely reflects incomplete inosculation and neovascularization • Improved take correlated with increased angiogenesis and decreased inflammation • Mixed graft models: allogenic (4) vs. autologous (1) — differing immunological dynamics • Species: 4 rodent, 1 miniature pig; variable exosome source, dose, and timing • Meta-analysis not performed due to high clinical heterogeneity	**Low***Small k; high heterogeneity; mixed graft models; publication bias cannot be excluded*

Abbreviations: k = number of studies; SMD = standardized mean difference; HPF = high-power field; IHC = immunohistochemistry; ELISA = enzyme-linked immunosorbent assay; ICG = indocyanine green; TUNEL = terminal deoxynucleotidyl transferase dUTP nick end labeling; HIF-1α = hypoxia-inducible factor 1-alpha; VEGF(A) = vascular endothelial growth factor (A); PI3K/AKT = phosphoinositide 3-kinase/protein kinase B; Bcl-2 = B-cell lymphoma 2.

Note: Meta-analysis was not performed for these secondary outcomes due to insufficient study numbers (*k* < 10) for reliable between-study variance estimation and small-study effect testing. Certainty ratings are informal assessments based on study consistency, sample sizes, risk of bias, and outcome heterogeneity; formal GRADE assessment was not applied, as GRADE is designed for clinical rather than preclinical evidence. Effect ranges are reported as individual study-level estimates, not pooled.

### Skin graft outcomes

Five studies evaluated skin graft survival: four in rodent models and one in miniature pigs (Table 2, Table 6). Four studies used allogeneic grafts and one used autologous grafts. At 7 days post-operatively, 2 of 5 studies reported significant improvements with exosome treatment; the remaining 3 showed no significant separation. By days 10–14, 4 of 5 studies demonstrated significantly improved graft survival. Day-14 survival of exosome-treated grafts ranged from 78 to 92% compared with 38–55% in controls (*p* < 0.05 in 4 studies). One study reported a non-significant difference (72%vs. 60%). Improved graft take correlated with increased angiogenesis and decreased inflammation. Considerable heterogeneity was evident; publication bias cannot be excluded.

## Discussion

Across 24 preclinical studies, exosome therapy was associated with directionally favourable changes in flap survival, angiogenesis, perfusion, VEGF expression, apoptosis, and skin graft take. In flap studies, meta-analysis suggested higher survival (MD 35.54%; 95% CI 25.11–45.97) and greater angiogenesis (SMD 3.60; 95% CI 2.66–4.54) after exosome treatment. These estimates should be interpreted cautiously. Funnel plot asymmetry and significant Egger's regression tests for both primary outcomes (flap survival *p* < 0.001; angiogenesis *p* < 0.001) indicate substantial small-study effects, and the convergence of small-study effects, extreme heterogeneity, and unclear methodological safeguards together suggests that the true treatment effect is likely meaningfully smaller than these pooled values. The findings of this review should therefore be read as a directionally consistent biological signal rather than as a quantitative estimate of clinical benefit.

Between-study heterogeneity was extreme across both primary endpoints (I² 76–97%), which fundamentally limits the interpretability of the pooled estimates. An I² of 97% for flap survival means that almost all observed variation between studies reflects true differences in underlying effects rather than sampling error; in practical terms, the studies are not estimating a single common treatment effect, and the pooled mean difference should be regarded as a mathematical average of widely divergent biological scenarios rather than as a generalisable estimate of "the effect of exosome therapy."

This dispersion likely reflects differences in flap model, ischaemia protocol, dose, exosome source, conditioning method, delivery route, carrier or scaffold use, follow-up time, and outcome measurement. Subgroup analyses by cellular source and species origin did not resolve this heterogeneity, and the limited number of studies per subgroup precluded meta-regression. The use of SMD for angiogenesis adds further caution because small within-study variance in tightly controlled animal experiments can produce large standardised effects.

Methodological safeguards were poorly reported across the evidence base, and this represents a major limitation rather than a neutral observation. Allocation concealment was unclear in all 24 studies (100%), random housing was unclear in 20 (83%), and blinding of caregivers/investigators and outcome assessors was unclear in 18 (75%) each. We emphasise that "unclear" is not equivalent to "low risk": it reflects an absence of reporting that prevents readers from determining whether the safeguard was implemented at all. Empirical evidence from preclinical research demonstrates that inadequate or unreported allocation concealment and blinding are systematically associated with inflated treatment effects, and this association almost certainly contributes to the magnitude of the pooled estimates and to the funnel plot asymmetry observed here.

We have not attempted to quantify a "corrected" estimate, because the trim-and-fill procedure imputed zero studies and any single-method correction would itself be unreliable in the presence of this much heterogeneity; we instead urge readers to treat all numerical estimates in this review as upper bounds rather than central estimates.

The biological mechanisms through which exosomes improve flap survival involve coordinated multi-pathway effects. Exosomes orchestrate choke vessel remodelling and neovascularization through the HIF-1α/VEGFA axis, identified in six studies.^4^^,^^6^^,^^39^^,^^40^^,^^43^^,^^48^ PI3K/AKT-mediated anti-apoptosis was reported in seven studies.^4^, ^5^, ^6^^,^^46^^,^^50^^,^^51^^,^^55^ Exosomes shift the flap microenvironment from pro-inflammatory to anti-inflammatory by reducing TNF-α, IL-1β, IL-6, and iNOS while increasing IL-10 through M1-to-M2 macrophage polarisation.^6^^,^^47^^,^^51^^,^^55^ This is significant because chronic or excessive inflammation leads to a cycle of compromised perfusion, ischaemia, cell damage, and further inflammatory amplification.^6^^,^^47^^,^^51^^,^^55^ Exosomes also inhibit PANoptosis, a pathological process that precedes vascular endothelial dysfunction and eventual flap necrosis. Activation of Keap1/Nrf2 pathways regulates apoptotic molecules such as BAD, Bax, caspase-3 and −9 while increasing antiapoptotic Bcl-2.^4^^,^^5^^,^^46^^,^^55^ BMSC-exosomes activated the Keap1/Nrf2 pathway to reduce reactive oxygen species and malondialdehyde while upregulating antioxidants SOD2 and catalase.^34^^,^^40^^,^^55^

Several miRNAs were implicated across studies: miR-132 in ADSC-exosomes increases new and mature blood vessels with functional networks,^39^ miR-126 contributes to endothelial cell proliferation and vascular stability,^42^ and miR-21, −130, −210, −421–3p, and −183–5p exert positive effects, though no single miRNA was uniformly investigated across multiple studies.^4^^,^^36^^,^^39^^,^^42^^,^^50^

Regarding cellular conditioning, hypoxia-preconditioned exosomes consistently demonstrated larger effect sizes, suggesting enriched pro-angiogenic cargo.^4^^,^^5^^,^^36^^,^^38^^,^^48^ Oxidative stress-preconditioned (H₂O₂) exosomes showed enhanced angiogenic properties via Wnt/β-catenin signalling in endothelial progenitor cells, recruiting circulating progenitors to ischaemic zones.^36^^,^^40^^,^^42^ LPS preconditioning of BMSC-exosomes shifted macrophage polarisation via NF-κB/NLRP3 suppression.^53^ These findings suggest that exosome cargo and therapeutic potency are significantly influenced by the parent cell type and conditioning environment, supporting exploration of bioengineered or preconditioned exosomes in future translational studies.

Skin graft outcomes were directionally favourable but based on only 5 studies with low certainty. The time-dependent improvement in graft take (significant from day 10–14) aligns with inosculation and revascularization timelines,^27^, ^28^, ^29^ suggesting exosomes accelerate vessel in-growth rather than producing immediate haemodynamic rescue.^16^^,^^55^ Exosomes exhibited protective effects on autologous grafts and prolonged survival of allogeneic grafts.^52^, ^53^, ^54^, ^55^, ^56^ However, the small number of studies, significant heterogeneity (mixed graft types, variable timepoints), and inability to perform meta-analysis limit certainty.

Exploratory subgroup data suggested that human ADSC-derived exosomes were associated with superior outcomes, consistent with literature indicating higher pro-angiogenic miRNA secretion and more robust paracrine effects from ADSCs.^16^^,^^39^^,^^42^ Adipose tissue is more accessible than bone marrow, making ADSC-exosome production more scalable. Human ADSC-derived exosomes may represent a reasonable priority for further standardised preclinical evaluation before any clinical consideration. Meta-regression on dose, timing, and delivery route could not be performed due to inconsistent reporting.

The clinical value proposition may differ between settings. In complex reconstruction (irradiated fields, high ischaemia burden, salvage scenarios), even modest improvements in survival could justify costs and logistics. In aesthetic surgery, where baseline complication rates are lower, the risk-benefit threshold is higher.^57^, ^58^, ^59^, ^60^ Any potential adoption would require standardised manufacturing/characterisation, regulatory approval, and robust safety and cost-effectiveness evidence. Exosomes have not been approved by any regulatory authority for clinical treatments; early adoption will require trial-grade manufacturing and transparent characterisation.

Several limitations must be acknowledged. Twenty-three of 24 studies used small rodent models; only one used miniature pigs. Most models used young, healthy animals, which may overestimate efficacy compared with surgical patients who frequently have diabetes, smoking exposure, vascular disease, or prior radiation. Rodent skin anatomy, perfusion dynamics, and immune responses differ from humans; therefore, these findings should be interpreted as biologic proof-of-concept rather than clinical efficacy. Heterogeneous reporting prevented dose-response analysis. All studies focused on early survival; long-term durability remains unknown. Substantial heterogeneity across exosome source, isolation, characterisation, dose, route, timing, and outcome measurement was evident. Risk-of-bias assessment revealed unclear allocation concealment and blinding in most studies, with frequent unclear ratings in critical domains potentially contributing to inflated effect sizes. Despite these limitations, the directionally similar signals across several related outcomes supports continued investigation, although the magnitude and reproducibility of benefit remain uncertain.

## Conclusion

Exosome therapy shows directionally favourable but preliminary preclinical signals for improving flap and skin graft survival. In animal models, exosomes were associated with better flap survival, angiogenesis, perfusion, VEGF expression, reduced apoptosis, and delayed improvement in skin graft take; however, these findings must be interpreted cautiously. Substantial heterogeneity, funnel plot asymmetry, significant Egger’s tests, and frequent unclear reporting of allocation concealment and blinding limit confidence in the pooled estimates and suggest that the true effect size may be smaller than observed. At present, the evidence should be regarded as exploratory and hypothesis-generating rather than clinically confirmatory. Future work should prioritise standardised exosome characterisation, more rigorous and reproducible preclinical study design, and carefully designed early-phase comparative clinical studies.

## Funding

None.

## Ethical approval

Not required.

## Declaration of AI and AI-assisted technologies in the writing process

During the preparation of this work the authors used Claude (Anthropic) in order to improve the readability, structure, and language of the manuscript during the revision process. All study design, literature search, data extraction, statistical analysis, and scientific interpretation were performed exclusively by the human authors. After using this tool, the authors reviewed and edited the content as needed and take full responsibility for the content of the publication.

## Conflicts of interest

None declared.

## Appendix Supplementary materials

Image, application 1

Image, application 2

Image, application 3

Image, application 4

## References

[1] Tan F., Li X., Wang Z., Li J., Shahzad K., Zheng J. (2024). Clinical applications of stem cell-derived exosomes. Sig Transduct Target Ther.

[2] Ku Y.C., Omer Sulaiman H., Anderson S.R., Abtahi A.R (2023). The potential role of exosomes in aesthetic plastic surgery: a review of current literature. Plast Reconstr Surg - Glob Open.

[3] Welsh J.A., Goberdhan D.C.I., O’Driscoll L. (2024). Minimal information for studies of extracellular vesicles (MISEV2023): from basic to advanced approaches. J Extracell Vesicle.

[4] Deng C., Dong K., Liu Y. (2023). Hypoxic mesenchymal stem cell-derived exosomes promote the survival of skin flaps after ischaemia-reperfusion injury via mTOR/ULK1/FUNDC1 pathways. J Nanobiotechnology.

[5] Liu Z., Chen D-H, Lin Z.-H. (2025). In-situ sprayed platelet-derived small extracellular vesicles for the skin flap survival by reducing PANoptosis. Biomaterials.

[6] Luo G., Zhou Z., Cao Z. (2024). M2 macrophage-derived exosomes induce angiogenesis and increase skin flap survival through HIF1AN/HIF-1α/VEGFA control. Arch Biochem Biophys.

[7] Couch Y., Buzàs E.I., Di Vizio D. (2021). A brief history of nearly EV-erything – The rise and rise of extracellular vesicles. J Extracell Vesicle.

[8] Gurung S., Perocheau D., Touramanidou L., Baruteau J. (2021). The exosome journey: from biogenesis to uptake and intracellular signalling. Cell Commun Signal.

[9] Bai G., Truong T.M., Pathak G.N., Benoit L., Rao B. (2024). Clinical applications of exosomes in cosmetic dermatology. Skin Health Dis.

[10] Chen Y.F., Luh F., Ho Y.S., Yen Y. (2024). Exosomes: a review of biologic function, diagnostic and targeted therapy applications, and clinical trials. J Biomed Sci.

[11] De Francesco F., Zingaretti N., Parodi P.C., Riccio M. (2023). The evolution of current concept of the reconstructive ladder in plastic surgery: the emerging role of translational medicine. Cells.

[12] Grosu-Bularda A., Hodea F.V., Cretu A. (2024). Reconstructive paradigms: a problem-solving approach in complex tissue defects. JCM.

[13] McGraw I.T., Wilson E.E., Behfar A., Paradise C.R., Rohrich R.J., Wyles S.P. (2024). Evolving role of exosomes in plastic and reconstructive surgery and dermatology. Plast Reconstr Surg - Glob Open.

[14] Prasai A., Jay J.W., Jupiter D., Wolf S.E., El Ayadi A. (2022). Role of exosomes in dermal wound healing: a systematic review. J Investig Dermatol.

[15] Ye H., Wang F., Xu G., Shu F., Fan K., Wang D. (2023). Advancements in engineered exosomes for wound repair: current research and future perspectives. Front Bioeng Biotechnol.

[16] Zhou C., Zhang B., Yang Y. (2023). Stem cell-derived exosomes: emerging therapeutic opportunities for wound healing. Stem Cell Res Ther.

[17] Manzoor T., Farooq N., Sharma A. (2024). Exosomes in nanomedicine: a promising cell-free therapeutic intervention in burn wounds. Stem Cell Res Ther.

[18] Wang M., Zhao X., Cui Y. (2025). Extracellular vesicles in burn injury: roles, mechanisms, and applications. Burns Trauma.

[19] Ghiasloo M., De Wilde L., Singh K. (2021). A systematic review on extracellular vesicles-enriched fat grafting: a shifting paradigm. Aesthet Surg J.

[20] Zhang Y., Liu T. (2023). Adipose-derived stem cells exosome and its potential applications in autologous fat grafting. J Plast Reconstr Aesthet Surg.

[21] Ersan M., Ozer E., Akin O., Tasli P.N., Sahin F. (2024). Effectiveness of exosome treatment in androgenetic alopecia: outcomes of a prospective study. Aesth Plast Surg.

[22] Norouzi F., Aghajani S., Vosoughi N. (2024). Exosomes derived stem cells as a modern therapeutic approach for skin rejuvenation and hair regrowth. Regen Ther.

[23] Ogawa M., Udono M., Teruya K., Uehara N., Katakura Y. (2021). Exosomes derived from fisetin-treated keratinocytes mediate hair growth promotion. Nutrients.

[24] Li C., Wei S., Xu Q., Sun Y., Ning X., Wang Z. (2022). Application of ADSCs and their exosomes in scar prevention. Stem Cell Rev Rep.

[25] Ogawa R. (2022). The most current algorithms for the treatment and prevention of hypertrophic scars and keloids: a 2020 update of the algorithms published 10 years ago. Plast Reconstr Surg.

[26] Zhong Y., Zhang Y., Yu A. (2023). Therapeutic role of exosomes and conditioned medium in keloid and hypertrophic scar and possible mechanisms. Front Physiol.

[27] Chung K.C., Gatsidis G., Sasor S.E. (2025).

[28] Neligan P.C., Mazzola R.F., Mazzola I.C. (2024).

[29] Kay S., Wilks D., McCombe D. (2021).

[30] Gimenez A.R., Rohrich R., Borab Z., Fisher S., Fagien S., Rohrich R.J. (2025). Safety and complications in lower eyelid blepharoplasty: a systematic review. Plast Reconstr Surg Glob Open.

[31] Hashem A.M., Couto R.A., Surek C., Swanson M., Zins J.E. (2021). Facelift Part II: surgical techniques and complications. Aesthet Surg J.

[32] Singhal S., Tobin V., Hunter-Smith D., Rozen W. (2024). Classification of postoperative complications in plastic and reconstructive surgery: a systematic review. Australas J Plast Surg.

[33] Bonomi F., Harder Y., Treglia G., De Monti M., Parodi C. (2024). Is free nipple grafting necessary in patients undergoing reduction mammoplasty for gigantomastia? A systematic review and meta-analysis. J Plast Reconstr Aesthet Surg.

[34] Bustos S.S., Kuruoglu D., Yan M. (2021). Nipple-areola complex reconstruction in transgender patients undergoing mastectomy with free nipple grafts: a systematic review of techniques and outcomes. Ann Transl Med.

[35] Tao B.K., Dhivagaran T., Butt F.R. (2025). Ectropion repair techniques and the role of adjunctive superotemporal skin transposition for tarsal Ectropion. JCM.

[36] Bai Y., Han Y.D., Yan X.L. (2018). Adipose mesenchymal stem cell-derived exosomes stimulated by hydrogen peroxide enhanced skin flap recovery in ischemia-reperfusion injury. Biochem Biophys Res Commun.

[37] Chang R., Wang P., Chen H. (2025). Multifunctional Hydrogel integrated hemangioma stem cell-derived nanovesicle-loaded metal-polyphenol network promotes diabetic flap survival. Adv Heal Mater.

[38] Ding J.P., Sun Y., Chen B., Qian W.J., Bao S.W., Zhao H.Y. (2024). Sequential transplantation of exosomes and BMSCs pretreated with hypoxia efficiently facilitates perforator skin flap survival area in rats. Br J Oral Maxillofac Surg.

[39] Ge L., Wang K., Lin H. (2023). Engineered exosomes derived from miR-132-overexpresssing adipose stem cells promoted diabetic wound healing and skin reconstruction. Front Bioeng Biotechnol.

[40] Guo L., Chen Y., Feng X. (2022). Oxidative stress-induced endothelial cells-derived exosomes accelerate skin flap survival through Lnc NEAT1-mediated promotion of endothelial progenitor cell function. Stem Cell Res Ther.

[41] Liu X., Chen H., Lei L. (2024). Exosomes-carried curcumin based on polysaccharide hydrogel promote flap survival. Int J Biol Macromol.

[42] Mayo J.S., Kurata W.E., O'Connor K.M., Pierce L.M. (2019). Oxidative stress alters angiogenic and antimicrobial content of extracellular vesicles and improves flap survival. Plast Reconstr Surg Glob Open.

[43] Ngo N.H., Chang Y.H., Vuong C.K. (2022). Transformed extracellular vesicles with high angiogenic ability as therapeutics of distal ischemic tissues. Front Cell Dev Biol.

[44] Niu Q., Yang Y., Li D. (2022). Exosomes derived from bone marrow mesenchymal stem cells alleviate ischemia-reperfusion injury and promote survival of skin flaps in rats. Life.

[45] Pu C.M., Liu C.W., Liang C.J. (2017). Adipose-derived stem cells protect skin flaps against ischemia/reperfusion injury via IL-6 expression. J Invest Dermatol.

[46] Shi X., Yang G., Liu M. (2023). Exosomes derived from Human dental pulp stem cells increase flap survival with ischemia-reperfusion injuries. Regen Med.

[47] Sun C., Su J., Wang Z. (2025). Engineering stem cell exosomes promotes the survival of multi-territory perforator flap in diabetes via regulating anti-inflammatory and angiogenesis. Regen Biomater.

[48] Wu S., Hu X., Wang Z.H. (2022). Extracellular vesicles isolated from hypoxia-preconditioned adipose-derived stem cells promote hypoxia-inducible factor 1α–Mediated neovascularization of random skin flap in rats. Ann Plast Surg.

[49] Xie L., Wang J., Zhang Y. (2019). The effects of local injection of exosomes derived from BMSCs on random skin flap in rats. Am J Transl Res.

[50] Zhang X., Jiang X., Deng H. (2024). Engineering exosomes from fibroblast growth factor 1 pre-conditioned adipose-derived stem cells promote ischemic skin flaps survival by activating autophagy. Mater Today Bio.

[51] Zhu L., Niu Q., Li D. (2025). Bone marrow mesenchymal stem cells-derived exosomes promote survival of random flaps in rats through Nrf2-mediated antioxidative stress. J Reconstr Microsurg.

[52] Campos-Mora M., De Solminihac J., Rojas C. (2022). Neuropilin-1 is present on Foxp3+ T regulatory cell-derived small extracellular vesicles and mediates immunity against skin transplantation. J Extracell Vesicl.

[53] Li C., Guo F., Wang X. (2020). Exosome-based targeted RNA delivery for immune tolerance induction in skin transplantation. J Biomed Mater Res A.

[54] Li P., Cao L., Liu T., Lu X., Ma Y., Wang H. (2025). The effect of adipose-derived stem cell (ADSC)-exos on the healing of autologous skin grafts in miniature pigs. Int J Mol Sci.

[55] Li X., Wang X., Cai H., et al. Impact of exosomes derived from adipose stem cells on lymphocyte proliferation and phenotype in mouse skin grafts. Extracell vesicles circ nucl acids. 2025;6(1):141–157. doi: 10.20517/evcna.2024.52. 10.20517/evcna.2024.52 PMC11977351 40206795

[56] Zhang P., Wu P., Khan U.Z. (2023). Exosomes derived from LPS-preconditioned bone marrow-derived MSC modulate macrophage plasticity to promote allograft survival via the NF-κb/NLRP3 signaling pathway. J Nanobiotechn.

[57] Bouhadana G., ElHawary H., Alam P., Gilardino M.S. (2024). A procedure and complication-specific assessment of smoking in aesthetic surgery: a systematic review and meta-analysis. Plast Surg.

[58] Eytan D.F., Wang T.D. (2022). Complications in rhinoplasty. Clin Plast Surg.

[59] Sharif-Askary B., Carlson A.R., Van Noord M.G., Marcus J.R. (2020). Incidence of postoperative adverse events after rhinoplasty: a systematic review. Plast Reconstr Surg.

[60] Abu El Hawa A.A., Dekker P.K., Mizher R., Orra S., Fan K.L., Del Corral G. (2022). Utility of negative pressure wound therapy: raising the bar in chest masculinization surgery. Plast Reconstr Surg - Glob Open.

