# Supplementary material for: Exosome therapy for flap and skin graft survival: A systematic review and meta-analysis of preclinical evidence
Source: JPRAS Open. 2026 Jun 1;50:665–84. doi: 10.1016/j.jpra.2026.05.042 (PMC13311139; doi:10.1016/j.jpra.2026.05.042)
Supplement: Supplementary file 1 [file mmc1.docx]

**Supplement 1. Detailed Search Strategies**

*Enhancing Flap and Skin Graft Survival with Exosome Therapy: Systematic Review and Meta-Analysis*

**Overview**

Electronic searches were conducted in PubMed, OVID Medline, Scopus, Web of Science Core Collection, and Google Scholar from inception to 12 September 2025. Searches were executed between 12–15 September 2025. No language or date restrictions were applied. Search strategies were adapted to database-specific controlled vocabulary (e.g., MeSH) and syntax. The exact search strings used and the number of records retrieved per database are presented below.

**PubMed (n = 592)**

**Platform/Interface:** PubMed (National Library of Medicine)

**Date range:** Inception to 12 Sep 2025

**Search run date(s):** 12–15 Sep 2025

**Exact search string:**

| (("Exosomes"[Mesh] OR exosome*[tiab] OR "extracellular vesicle*"[tiab] OR "extracellular vesicles"[Mesh] OR EV[tiab] OR EVs[tiab] OR "small extracellular vesicle*"[tiab] OR sEV[tiab] OR sEVs[tiab] OR "microvesicle*"[tiab])) AND (("Surgical Flaps"[Mesh] OR flap*[tiab] OR "skin flap*"[tiab] OR "cutaneous flap*"[tiab] OR "random pattern flap*"[tiab] OR "axial flap*"[tiab] OR "Skin Transplantation"[Mesh] OR graft*[tiab] OR "skin graft*"[tiab] OR "split thickness skin graft*"[tiab] OR "full thickness skin graft*"[tiab])) AND ((animals[mh] OR animal*[tiab] OR murine[tiab] OR mouse[tiab] OR mice[tiab] OR rat[tiab] OR rats[tiab] OR rabbit*[tiab] OR porcine[tiab] OR swine[tiab] OR "in vivo"[tiab]) NOT (humans[mh])) |
| --- |

**Notes:**

- No filters for article type, language, or publication date were applied.
- The animals-only restriction was implemented using PubMed indexing terms (animals[mh]) and exclusion of humans[mh].

**OVID Medline (n = 1490)**

**Platform/Interface:** Ovid MEDLINE(R) (via OVID)

**Date range:** Inception to 12 Sep 2025

**Search run date(s):** 12–15 Sep 2025

**Exact search string:**

| 1. exp Exosomes/ or exp Extracellular Vesicles/ or (exosome* or extracellular vesicle* or EV or EVs or sEV or sEVs or microvesicle*).mp. 2. exp Surgical Flaps/ or (flap* or skin flap* or cutaneous flap* or random pattern flap* or axial flap*).mp. 3. exp Skin Transplantation/ or (graft* or skin graft* or split thickness skin graft* or full thickness skin graft*).mp. 4. 2 or 3 5. exp Animals/ or (animal* or in vivo or murine or mouse or mice or rat or rats or rabbit* or porcine or swine).mp. 6. 1 and 4 and 5 7. remove duplicates from 6 |
| --- |

**Notes:**

- No limits were applied (language, year, publication type).
- Line 7 reflects Ovid’s interface-level duplicate removal within the database search results.
- Searched using both MeSH subject headings (exploded) and multi-purpose (.mp.) fields including title, abstract, keyword heading, and original title.
- Higher yield (n = 1,490) compared to PubMed reflects the broader .mp. field search and the exploded MeSH hierarchies in OVID.

**Scopus (n = 176)**

**Platform/Interface:** Scopus (Elsevier)

**Date range:** Inception to 12 Sep 2025

**Search run date(s):** 12–15 Sep 2025

**Exact search string:**

| TITLE-ABS-KEY(exosome* OR "extracellular vesicle*" OR EV OR EVs OR "small extracellular vesicle*" OR sEV OR sEVs OR microvesicle*) AND TITLE-ABS-KEY(flap* OR "skin flap*" OR "cutaneous flap*" OR "random pattern flap*" OR "axial flap*" OR graft* OR "skin graft*" OR "skin transplantation") AND TITLE-ABS-KEY("in vivo" OR animal* OR murine OR mouse OR mice OR rat OR rats OR rabbit* OR porcine OR swine) |
| --- |

**Notes:**

- No filters were applied for document type, language, or year.
- Scopus does not use MeSH/controlled vocabulary. Free-text keyword searching with wildcards (*) was used across title, abstract, and keyword fields.

**Web of Science (n = 460)**

**Platform/Interface:** Web of Science Core Collection

**Date range:** Inception to 12 Sep 2025

**Search run date(s):** 12–15 Sep 2025

**Exact search string:**

| TS=(exosome* OR "extracellular vesicle*" OR EV OR EVs OR "small extracellular vesicle*" OR sEV OR sEVs OR microvesicle*) AND TS=(flap* OR "skin flap*" OR "cutaneous flap*" OR "random pattern flap*" OR "axial flap*" OR graft* OR "skin graft*" OR "skin transplantation") AND TS=("in vivo" OR animal* OR murine OR mouse OR mice OR rat OR rats OR rabbit* OR porcine OR swine) |
| --- |

**Notes:**

- TS searches Topic fields (title, abstract, author keywords, and Keywords Plus).
- No filters were applied for document type, language, or year.
- All databases within the Core Collection were included (SCI-EXPANDED, SSCI, ESCI).

**Google Scholar (n = 21)**

**Platform/Interface:** Google Scholar

**Date range:** Inception to 12 Sep 2025

**Search run date(s):** 12–15 Sep 2025

**Exact search string:**

| "exosome" OR "extracellular vesicle" OR "small extracellular vesicle" AND "skin flap" OR "surgical flap" OR "skin graft" OR "skin transplantation" AND animal OR "in vivo" OR rat OR mouse OR murine OR rabbit |
| --- |

**Notes:**

- Google Scholar was searched using the query above; results were sorted by relevance.
- The first 200 results were screened, and potentially relevant records were retrieved for full-text evaluation.
- This database served as a supplementary source to capture any studies potentially missed by the primary databases.
- Only 21 unique records not already identified through the other databases were retrieved.

**Additional sources:**

Reference lists of included studies and relevant reviews were hand-searched to identify additional eligible studies.
