# Supplementary material for: Exosome therapy for flap and skin graft survival: A systematic review and meta-analysis of preclinical evidence
Source: JPRAS Open. 2026 Jun 1;50:665–84. doi: 10.1016/j.jpra.2026.05.042 (PMC13311139; doi:10.1016/j.jpra.2026.05.042)
Supplement: Supplementary file 2 [file mmc2.docx]

**Supplementary Table 2.** Exosome Isolation, Characterization, and Secondary Outcomes of Flap Studies

| **Study** | **Isolation Method** | **Characterization Methods & Markers** | **Particle Size, Concentration & Volume** | **Control Group** | **Secondary Outcomes (Exosome vs. Control)** |
| --- | --- | --- | --- | --- | --- |
| **Bai et al., 2018**^36^ | Ultracentrifugation (110,000 g) | TEM, NTA, WB (CD9, CD63, TSG101) | 75 nm; 6.65×10⁸ particles/mL; 200 µL | PBS (200 µL; n=6) | Perfusion: Exo 108,571 ± 12,725 / Ctrl 58,701 ± 22,904  Apoptosis: Exo 16,667 ± 4,474 / Ctrl 91,553 ± 6,711 |
| **Chang et al., 2025**^37^ | Ultracentrifugation (20,000 rpm) | TEM, NTA, WB (TSG101, CD9, CD63) | 116.7 ± 2.9 nm; 5×10⁶ cells/mL; 150 µL | PBS (150 µL; n=3) | — |
| **Deng et al., 2023**^4^ | ExoQuick-TC precipitation | TEM, NTA, WB (CD9, CD63) | 115.3 nm; 200 µg | PBS | VEGF: Exo 0.827 ± 0.142 / Ctrl 0.297 ± 0.142  Apoptosis: Exo 2,129 ± 2,689 / Ctrl 31,373 ± 6,275 |
| **Ding et al., 2024**^38^ | Ultracentrifugation (110,000 g) | TEM, NTA, WB (TSG101, CD9, CD63, CD81) | 121.4 nm; 2×10⁶ CM-Dil; 100 µL | PBS (100 µL; n=3) | — |
| **Ge et al., 2023**^39^ | Ultracentrifugation (120,000 g) | TEM, NTA, WB (CD9, CD63, CD81, TSG101) | 120 nm; 1×10¹⁰ particles/mL; 100 µL | PBS (100 µL; n=6) | Perfusion: Exo 446,389 ± 21,882 / Ctrl 216,630 ± 75,492 |
| **Guo et al., 2022**^40^ | Ultracentrifugation (120,000 g) | TEM, NTA, WB (CD9, CD63, CD81, TSG101) | 109.9 nm; 500 µg; 200 µL | PBS (200 µL; n=5) | VEGF: Exo 1.451 ± 0.347 / Ctrl 0.972 ± 0.181 |
| **Liu et al., 2024**^41^ | Ultracentrifugation (110,000 g) | TEM, NTA, WB (TSG101, CD9, CD63) | 120 nm; 32 µg | PBS (n=6) | — |
| **Liu et al., 2025**^5^ | Ultracentrifugation (100,000 g, 70 min) | DLS, NTA, TEM, WB (TSG101, CD9, CD63), Zeta potential | 100–200 nm; 2.09×10¹⁰ particles/mL; 100 µL | Saline (100 µL; n=8) | Apoptosis: Exo 26,168 ± 5,607 / Ctrl 83,879 ± 6,776 |
| **Luo et al., 2024**^6^ | Ultracentrifugation (100,000 g, 60 min) | TEM, DLS, WB (CD63, TSG101) | 100 nm; 500 µg; 100 µL | PBS (100 µL; n=6) | VEGF: Exo 2.058 ± 0.489 / Ctrl 0.557 ± 0.299 |
| **Mayo et al., 2019**^42^ | ExoQuick-TC Ultra precipitation | NTA | 150 ± 29 nm; 3×10¹⁰ particles; 300 µL | Vehicle (300 µL; n=5) | — |
| **Ngo et al., 2022**^43^ | Ultracentrifugation (140,000 g) | TEM, WB (TSG101, CD40, integrin β1) | 100 nm; 60 µg; 80 µg/10⁶; 100 µL | PBS (100 µL; n=9) | VEGF: Exo 2.724 ± 0.638 / Ctrl 0.984 ± 0.128 |
| **Niu et al., 2022**^44^ | ExoQuick-TC precipitation | TEM, NTA, WB (Alix, CD9, CD63) | 148 nm; 500 µg; 1 mg/mL; 500 µL | PBS (500 µL; n=6) | VEGF: Exo 1.065 ± 0.115 / Ctrl 0.507 ± 0.295  Apoptosis: Exo 10,328 ± 6,885 / Ctrl 25,279 ± 6,885 |
| **Pu et al., 2017**^45^ | ExoQuick-TC precipitation | TEM, WB (CD63, IL6) | 60–200 nm; 100 µg/mL; 1×10⁶; 120 µL | Saline (120 µL; n=6) | — |
| **Shi et al., 2023**^46^ | Ultracentrifugation (100,000 g) | TEM, NTA, WB (CD9, HSP70, TSG101) | 117.9 nm; 100 µg; 1×10⁵; 200 µL | PBS (200 µL; n=12) | Apoptosis: Exo 24,083 ± 7,798 / Ctrl 73,853 ± 16,055 |
| **Sun et al., 2025**^47^ | Ultracentrifugation (100,000 g) | TEM, DLS, WB (CD9, CD63, TSG101) | 30–200 nm; 1×10⁵; 200 µL | PBS (200 µL; n=4) | Perfusion: Exo 690,732 ± 97,561 / Ctrl 282,927 ± 103,415 |
| **Wu et al., 2022**^48^ | Ultracentrifugation | TEM, NTA, WB | 158.0 ± 32.6 nm; 10 µg; 50 µg/mL; 0.2 mL | PBS (0.2 mL; n=10) | Perfusion: Exo 803,200 ± 24,300 / Ctrl 354,700 ± 20,900  VEGF: Exo 11,056 ± 2,484 / Ctrl 3,447 ± 1,118 |
| **Xie et al., 2019**^49^ | Commercial exosome extraction kit | TEM, WB (CD9, CD63, TSG101) | 80–100 nm; 135 µg; 50 µg/mL; 2.7 mL | PBS (2.7 mL; n=15) | Perfusion: Exo 124,570 ± 41,750 / Ctrl 76,210 ± 15,460  VEGF: Exo 5,577 ± 1,352 / Ctrl 3,070 ± 1,690 |
| **Zhang et al., 2024**^50^ | Ultracentrifugation (100,000 g) | TEM, NTA, WB (CD81, CD9) | 100–150 nm; 100 µg; 100 µg/mL; 100 µL | PBS (100 µL; n=5) | Perfusion: Exo 360,671 ± 10,725 / Ctrl 218,705 ± 42,898 |
| **Zhu et al., 2024**^51^ | ExoQuick-TC precipitation | TEM, NTA, WB (CD9, CD63, CD81, Alix) | 50–100 nm; 200 µg; 1 µg/µL; 200 µL | PBS (200 µL; n=6) | Perfusion: Exo 172,464 ± 10,870 / Ctrl 155,072 ± 5,797  Apoptosis: Exo 5,128 ± 1,410 / Ctrl 16,603 ± 3,333 |

**Abbreviations:** TEM = transmission electron microscopy; NTA = nanoparticle tracking analysis; DLS = dynamic light scattering; WB = Western blot; VEGF = vascular endothelial growth factor; Exo = exosome group; Ctrl = control group. Surface markers: CD9, CD63, CD81, TSG101, Alix, HSP70 are standard EV markers per MISEV 2018/2023 guidelines.

**Note:** This table supplements simplified Table 1 in the main manuscript. Values for secondary outcomes are reported as mean ± SD. “—” indicates no secondary outcomes were reported for that study. Perfusion values are in arbitrary perfusion units. VEGF values represent relative protein expression or fold change as reported by individual studies. Apoptosis values represent apoptotic index (TUNEL-positive or caspase-3-positive cells per field).
