# Supplementary figures and images for: Exosome therapy for flap and skin graft survival: A systematic review and meta-analysis of preclinical evidence
Source: JPRAS Open. 2026 Jun 1;50:665–84. doi: 10.1016/j.jpra.2026.05.042 (PMC13311139; doi:10.1016/j.jpra.2026.05.042)

Supplement 3. Leave-One-Out Flap Survival (Sun et al., 2025)

**
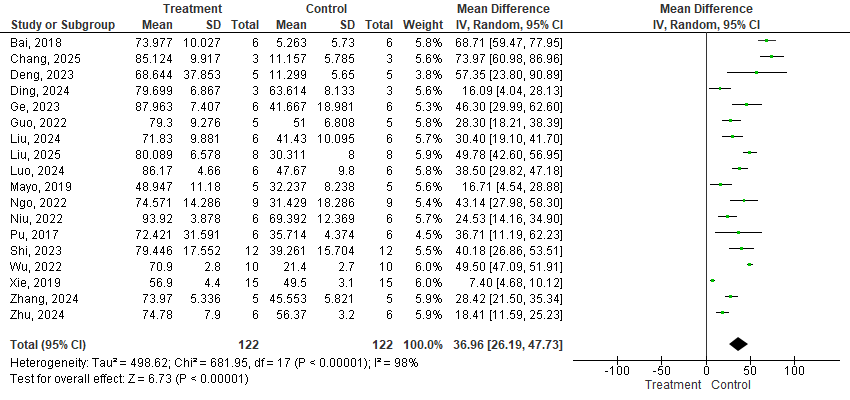
**

**
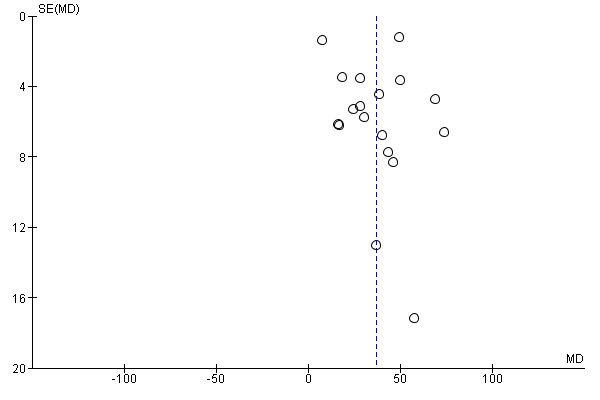
**

Supplement: Supplementary file 3 [file mmc3.docx]

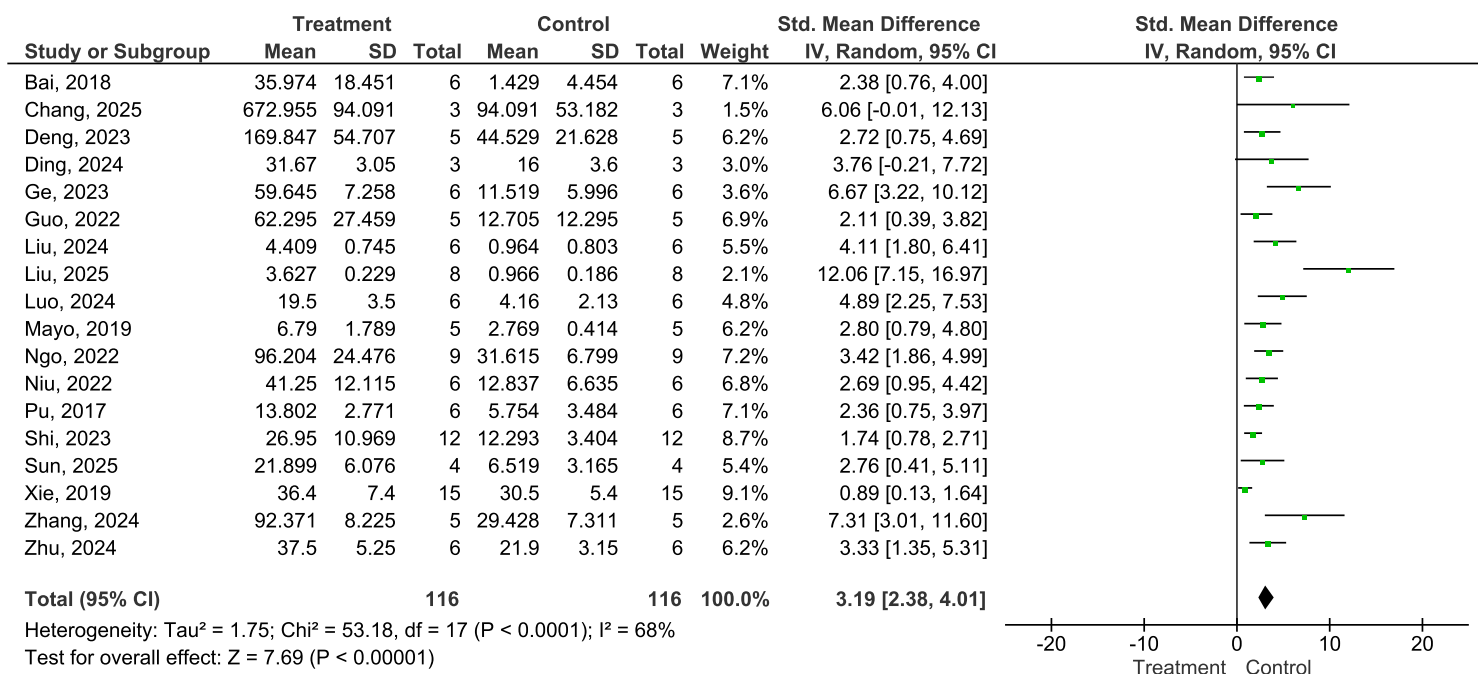

Supplement: Supplementary file 4 [file mmc4.pdf]
